# Evolution of Clinical Indications for Mitral Valve Transcatheter Edge-to-Edge Repair

**DOI:** 10.31083/RCM47139

**Published:** 2026-03-23

**Authors:** Carla Iglesias-Otero, Julio Echarte-Morales, David González-Fernández, Andrés Íñiguez-Romo, Rodrigo Estévez-Loureiro

**Affiliations:** ^1^Department of Cardiology, University Hospital Alvaro Cunqueiro, 36312 Vigo, Spain; ^2^Cardiovascular Research Group, Department of Cardiology, University Hospital Alvaro Cunqueiro, Fundación Biomédica Galicia Sur, Servizo Galego de Saude, University of Vigo, 36312 Vigo, Spain

**Keywords:** mitral regurgitation, mitral transcatheter edge-to-edge repair, heart failure, acute mitral regurgitation, expanding indications

## Abstract

Mitral valve transcatheter edge-to-edge repair (M-TEER) has evolved from a highly specialized intervention to an essential treatment option for patients with severe mitral regurgitation (MR) who are unsuitable candidates for surgery. Moreover, current guidelines support the use of M-TEER in both secondary MR and selected cases of primary MR. In addition to these established indications, data from clinical trials and registries indicate that M-TEER is associated with improved short-term outcomes compared with conservative therapy in acute MR after myocardial infarction, and is beneficial in more complex scenarios, such as advanced heart failure, hypertrophic obstructive cardiomyopathy, and mitral annulus calcification. Meanwhile, combined strategies, such as repairing the mitral and tricuspid valves simultaneously, adding M-TEER to transcatheter aortic valve replacement, or performing this procedure alongside left atrial appendage closure, are gaining ground as practical ways to address the broader needs of these high-risk patients. More recently, M-TEER has been used in patients with moderate MR, as this stage is now recognized to be associated with adverse outcomes. Overall, current evidence supports M-TEER as a safe and versatile therapy across an expanding range of clinical scenarios. Nonetheless, ongoing studies will help further clarify long-term outcomes and refine patient selection.

## 1. Introduction

Mitral regurgitation (MR) is commonly classified as primary (degenerative) or secondary (functional), depending on whether the primary abnormality involves the mitral valve apparatus itself or results from left ventricular remodeling and dysfunction. This distinction is clinically relevant, as underlying mechanisms, therapeutic strategies, and expected outcomes differ substantially between these entities.

Mitral valve repair surgery has traditionally been considered the first-line treatment for both primary and secondary MR, with high efficacy rates particularly in patients at low surgical risk [1, 2]. In recent years, mitral transcatheter edge-to-edge repair (M-TEER) has evolved from an emerging procedure to the therapeutic strategy of choice for patients with severe secondary MR and high surgical risk. The American Heart Association/American College of Cardiology guidelines assign M-TEER a class IIa indication for symptomatic patients with primary or secondary MR who are at high surgical risk or deemed inoperable [3], whereas the ESC guidelines provide a class I recommendation for patients with symptomatic secondary (ventricular) MR persisting despite guideline-directed medical therapy, and a class IIa recommendation for patients with primary MR who are symptomatic, have suitable anatomy, and are at high surgical risk [4]. This recommendation is supported by robust evidence showing that, compared with surgery, the percutaneous approach is associated with lower periprocedural morbidity, significant improvements in quality of life, and reductions in both heart failure (HF)–related hospitalizations and all-cause mortality [5].

Beyond established indications, M-TEER is increasingly being adopted across a broader range of clinical scenarios, reflecting expanding experience and encouraging outcomes in real-world practice. The purpose of this review is to investigate the various clinical scenarios in which the use of M-TEER has allowed for an expansion beyond established indications. These include settings in which M-TEER has been applied based on real-world experience and emerging evidence, such as acute ischemic MR, papillary muscle rupture (PMR), functional MR following myocardial infarction, moderate secondary MR, hypertrophic obstructive cardiomyopathy (HOCM), advanced HF, concomitant transcatheter interventions, and mitral annulus calcification.

## 2. Acute Mitral Regurgitation Following Acute Myocardial Infarction

**Fig. 1.**
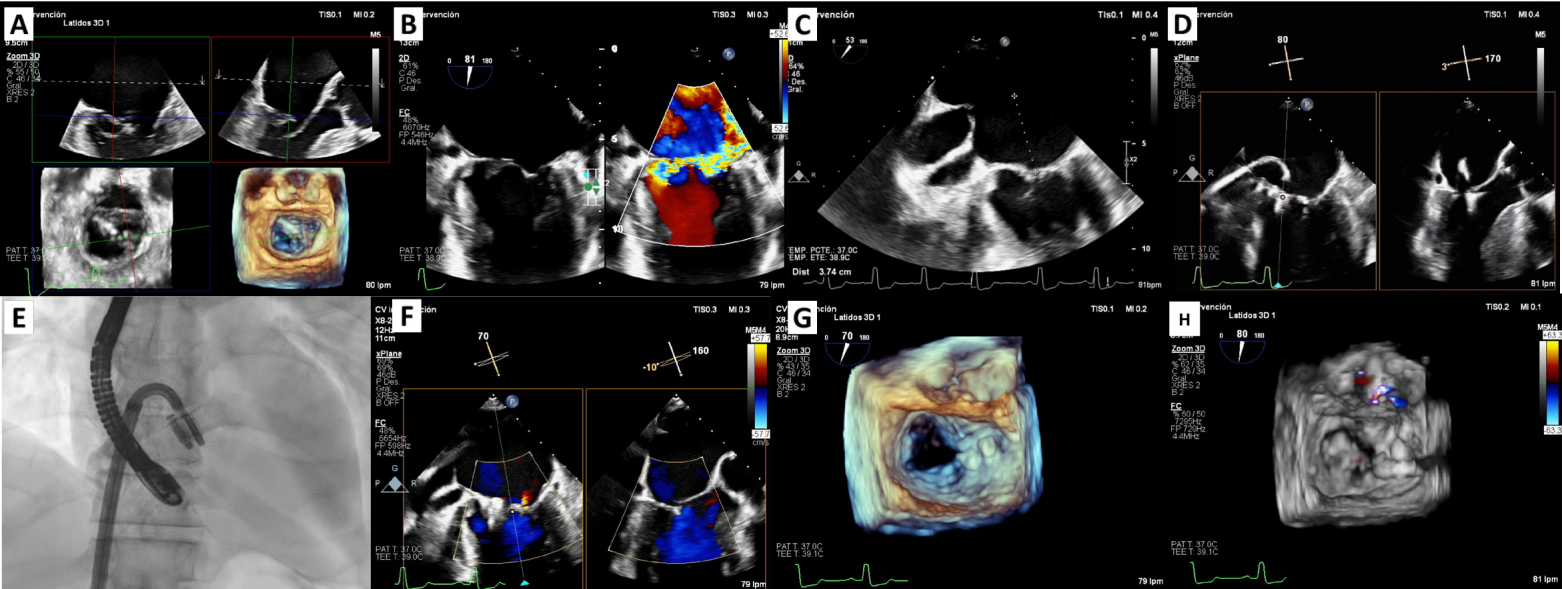
**M-TEER in acute ischemic mitral regurgitation**. (A) 3D multiplanar view of TEE showing chordal rupture and P3 scallop prolapse. (B) 2D TEE using orthogonal views: severe mitral regurgitation. (C) TEE: transseptal puncture at 37 mm height. (D) PASCAL Ace implantation: the first device was implanted in the medial commissural position and the second device in A3-P3 in close relation to the first one. (E) Fluoroscopy image of the implantation of two PASCAL Ace devices. (F) Mild mitral regurgitation post M-TEER. (G,H) Zoom-3D w/o colour: final result. M-TEER, mitral transcatheter edge-to-edge repair; TEE, transesophageal echocardiogram; W/o, with and without.

Acute MR is a serious complication of acute myocardial infarction (AMI) associated with high morbidity and mortality [6, 7] (Fig. 1). A recent large study found that most patients had mild MR (76%), followed by moderate (21%) and severe MR (3%) [8]. In the CADILLAC trial, which evaluated patients with AMI treated with primary percutaneous coronary intervention, MR was found in approximately 13% of patients at baseline ventriculography [9]. Even though most cases were mild, the presence and severity of MR were closely associated with older age, female sex, higher Killip class, multivessel coronary disease, and reduced left ventricular function. Importantly, MR of any grade emerged as an independent predictor of mortality at both 30 days and one year, with a stepwise increase in risk from mild to severe MR. These findings underscore that ischemic MR, even when mild, is not a benign finding in the context of AMI and continues to carry adverse prognostic significance despite timely mechanical reperfusion.

While PMR is the classical presentation described in the literature, MR may also manifest in a more “functional” phenotype, leading to marked clinical deterioration. Distinguishing between these mechanisms is critical for understanding the clinical presentation and guiding therapy. Mitral valve replacement surgery in this setting carries a high surgical risk, especially when compared to percutaneous repair in terms of morbidity and mortality 30 days post-intervention. A systematic review reported an aggregate 30-day mortality rate of 19%, with some studies describing rates approaching 40% during the acute phase [10]. In the current era of transcatheter mitral valve interventions, M-TEER has emerged as a percutaneous option for post-infarction acute MR. It should be noted that no randomized controlled trials have evaluated M-TEER in this setting; available evidence is limited to observational studies and case series. Some reports suggest that the procedure may improve survival in selected patients [11].

### 2.1 Papillary Muscle Rupture 

The use of percutaneous therapies is expanding, largely because a considerable proportion of patients with acute MR are not candidates for surgery. Decisions against surgery are often driven by advanced age, comorbidities, and hemodynamic instability. Even in those who undergo surgery, outcomes remain poor, with high early mortality and frequent complications such as transfusion needs, renal failure, and prolonged ventilation [12]. These challenges highlight the value of less invasive therapies to broaden treatment options for post-AMI patients with MR.

Recently, investigators have reported the largest series to date of patients with PMR after acute MI treated with M-TEER. Out of 655 patients in the registry, 23 presented with complete, partial, or chordal rupture [13]. This was an extremely high-risk cohort, with a median EuroSCORE II of 27%, 87% in cardiogenic shock, and nearly three-quarters requiring mechanical circulatory support. M-TEER was performed early (median 6 days post-MI), achieving procedural success in 87% of cases, with marked MR reduction and significant hemodynamic improvement, including a fall in left atrial V-wave (49 to 26 mmHg) and systolic pulmonary artery pressure (50 to 40 mmHg). Despite these encouraging results, in-hospital mortality remained 30%, reflecting the severity of the condition, yet 70% of patients survived to discharge, most with symptomatic improvement. Importantly, five patients subsequently underwent delayed surgical replacement, and no further deaths were observed among survivors at one year. These data suggest that M-TEER has been associated with improved short-term outcomes compared with conservative therapy

### 2.2 Secondary MR Phenotype 

The European Registry of MitraClip (Abbott) in Acute Mitral Regurgitation following Acute Myocardial Infarction (EREMMI) prospectively enrolled 44 patients with severe MR developing shortly after transmural MI who were deemed at prohibitive surgical risk [14]. Median EuroSCORE II was 15.1%, reflecting extreme risk. MitraClip implantation achieved technical success in 86.6%, with significant MR reduction and rapid clinical improvement. Thirty-day mortality was 9.1%, and at six months 72.5% of survivors had MR ≤2+ and 75.9% were in New York Heart Association (NYHA) class I–II. Despite the small sample size, this registry suggests the feasibility, safety, and meaningful clinical benefit of transcatheter edge-to-edge repair in acute post-MI MR.

The International Registry of MitraClip in Acute Mitral Regurgitation following Acute Myocardial Infarction (IREMMI) suggests the feasibility of M-TEER in this high-risk population, showing better outcomes compared with conservative management in post-AMI functional MR and supporting M-TEER as a viable alternative to surgery in selected patients [15]. Building on this, the registry recently reported outcomes from 471 patients across 21 centers. All had at least moderate-to-severe MR with HF symptoms within 90 days of MI and were treated with surgical mitral valve repair/replacement (SMVR), M-TEER (MitraClip or PASCAL [Edwards Lifesciences]) or conservative therapy. Of these, 205 underwent intervention (106 surgery, 99 TEER). Surgery was performed earlier than M-TEER (median 12 vs. 19 days post-AMI; *p* = 0.01). Despite worse baseline status, patients who underwent intervention had better outcomes than those managed conservatively (in-hospital mortality 11% vs. 27%; 1-year mortality 16% vs. 35%). Although procedural success was similar between surgery and M-TEER, the lower mortality with M-TEER suggests it may offer a safer alternative for high-risk post-AMI patients with significant MR.

**Fig. 2.**
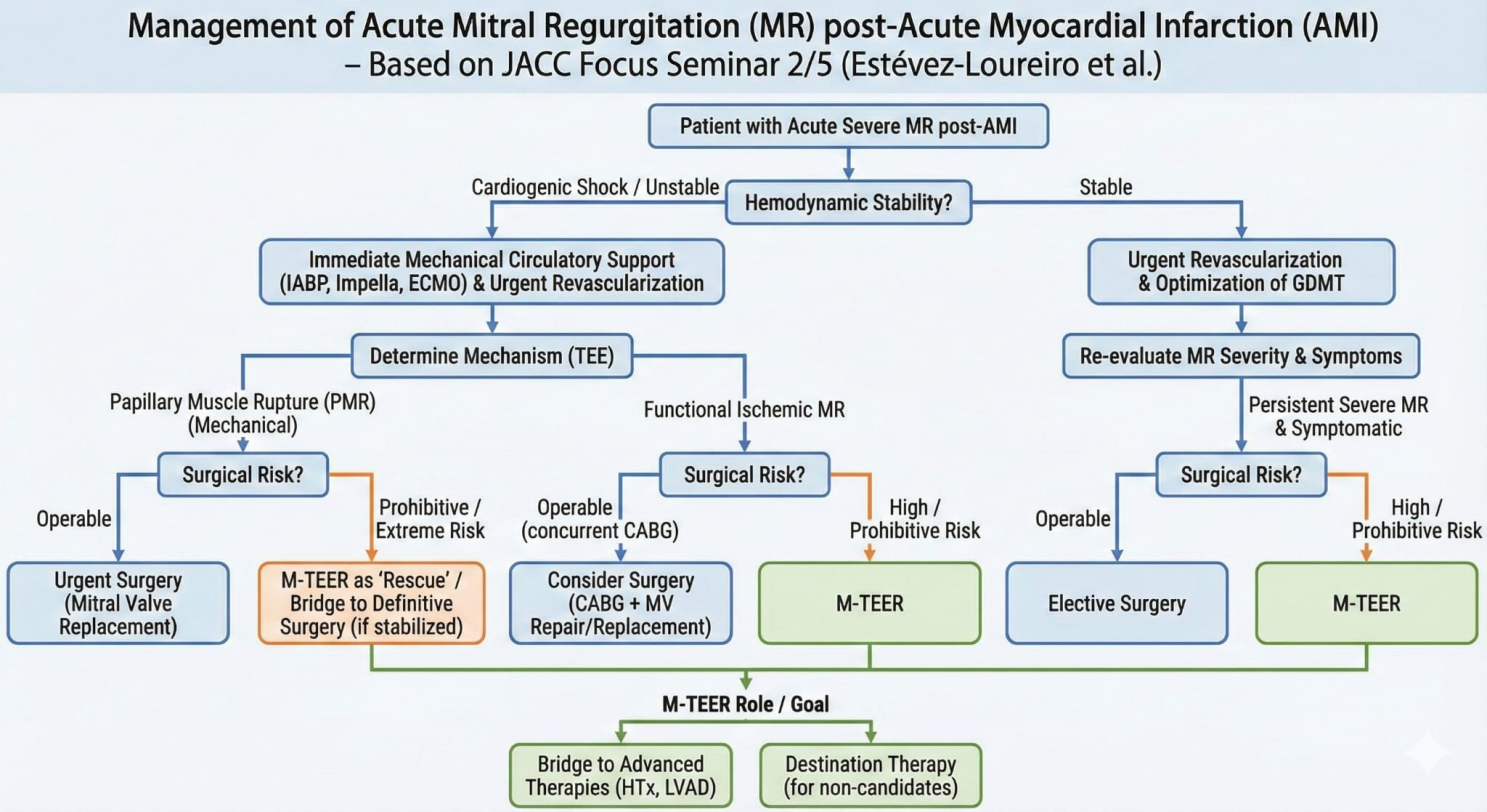
**Management algorithm for acute severe mitral regurgitation after myocardial infarction**. AMI, acute myocardial infarction; CABG, coronary artery bypass grafting; GDMT, guideline-directed medical therapy; MR, mitral regurgitation; M-TEER, mitral transcatheter edge-to-edge repair; MV, mitral valve; TEE, transesophageal echocardiogram; LVAD, left ventricular assist device; IABP, intra-aortic balloon pump; ECMO, extracorporeal membrane oxygenation.

Another subanalysis from the IREMMI Registry evaluated 93 patients with acute MR after AMI treated with MitraClip across 18 centers, of whom 54% presented with cardiogenic shock [16]. Procedural success was high and comparable between cardiogenic and non-cardiogenic shock patients (90% vs. 93%), with no excess in major complications. At 30 days, mortality was numerically higher in cardiogenic shock (10% vs. 2.3%), but not statistically significant, and after a median 7-month follow-up, combined death or HF rehospitalization did not differ between groups (28% vs. 26%). Multivariable analysis confirmed that cardiogenic shock was not independently associated with adverse outcomes, whereas procedural success strongly predicted survival. These findings support the feasibility of MitraClip in acute post-AMI MR even in patients presenting with CS, provided initial stabilization is achieved (Fig. 2).

## 3. Moderate Secondary Mitral Regurgitation 

Over the past decade, evidence supporting M-TEER has expanded considerably. Yet, two landmark trials—MITRA-FR and COAPT—reported strikingly different outcomes despite enrolling seemingly similar patient populations [5, 17]. In MITRA-FR, patients had symptomatic HF with left ventricular ejection fraction (LVEF) 15–40%, larger LV volumes (mean LVEDV 135 mL/m^2^), and only moderate-to-severe MR (effective regurgitant orifice area [EROA] ≥20 mm^2^; regurgitant volume ≥30 mL). The trial failed to demonstrate a benefit of MitraClip over optimal medical therapy. In contrast, COAPT enrolled patients with symptomatic HF and more severe MR (EROA ≥30 mm^2^, regurgitant volume >45 mL), smaller ventricles (LVEDV 101 mL/m^2^), and optimized medical and device therapy prior to enrollment. In this more “disproportionate” MR phenotype—where MR severity exceeded the degree of LV dilation—M-TEER significantly reduced both HF hospitalizations and all-cause mortality. These divergent results gave rise to the concept of proportionate versus disproportionate MR, which links regurgitation severity to LV remodeling [18, 19]. Patients with disproportionate MR (severe MR relative to LV size) appear most likely to benefit from M-TEER, whereas those with proportionate MR (moderate MR appropriate to severe LV dilation) derive limited hemodynamic improvement.

A subsequent randomized trial, RESHAPE-HF2, further expanded the evidence for M-TEER by including patients with HF and moderate-to-severe secondary MR across a broader range of LV systolic function, thereby extending the target population beyond the classical paradigm of secondary MR associated with severe LV dysfunction [20]. The study enrolled 505 patients with symptomatic HF, LVEF 20–50%, recent HF hospitalizations, and EROA >20 mm^2^, all on guideline-directed medical therapy. At 24 months, the addition of MitraClip to medical therapy significantly reduced the composite of first or recurrent HF hospitalizations or cardiovascular death (rate ratio 0.64, 95% CI 0.48–0.85; *p* = 0.002), as well as the rate of HF hospitalizations alone (rate ratio 0.59, 95% CI 0.42–0.82). Procedural success was high, with durable MR reduction in ~90% of patients, and health status markedly improved at 12 months compared with controls. Collectively, RESHAPE-HF2 confirmed the results of COAPT and extended the benefit of M-TEER to a broader population with moderate MR and less advanced LV dysfunction, reinforcing its role in reducing HF events and improving quality of life. This has prompted a reevaluation of conventional severity thresholds (e.g., EROA ≥40 mm^2^) and generated debate about earlier intervention in selected patients with less severe MR but persistent symptoms and inadequate left ventricular remodeling.

Asgar *et al*. [21] confirmed the importance of M-TEER in moderate secondary MR in a real-world population. The safety and effectiveness were evaluated in both moderate (335 patients) and severe MR (525 patients). There were no differences in baseline characteristics, and patients with moderate MR experienced significant 1-year improvements in NYHA functional class and quality of life (>20 points on the Kansas City Cardiomyopathy Questionnaire–Overall Summary). Additionally, significant LV reverse remodeling was observed, reflected by a >20 mL decrease in LV end-diastolic and end-systolic volumes in the moderate MR cohort. Rates of major adverse events were low in both groups, and the occurrence of death and HF hospitalizations was similar between the two subgroups.

Taken together, these data suggest that patients with symptomatic HF, clearly quantifiable moderate-to-severe MR, optimized medical and device therapy, and LV volumes that are not excessively remodeled represent the phenotype most likely to benefit from M-TEER.

## 4. Hypertrophic Obstructive Cardiomyopathy 

Hypertrophic cardiomyopathy (HCM) has an estimated prevalence of 1:200 to 1:500 in the general population [22]. Approximately 25% to 70% of patients develop dynamic left ventricular outflow tract (LVOT) obstruction, both at rest (25% to 30%) and when induced by Valsalva maneuvers (up to 70%). This is determined by the systolic anterior motion (SAM) of the mitral valve leaflets, which is responsible for LVOT obstruction and MR.

Pharmacological therapy remains the first-line treatment. Beta-blockers, non-dihydropyridine calcium channel blockers, and disopyramide have been traditionally used. However, recent studies have positioned cardiac myosin inhibitors (mavacamten or aficamten) as effective options in obstructive forms of HCM (EXPLORER-HCM and SEQUOIA-HCM) [23, 24]. They are also included in the latest ESC clinical practice guidelines with a class IIa recommendation [25]. In patients with a persistent LVOT gradient ≥50 mmHg, severe symptoms (NYHA class III–IV) and a history of syncope despite medical treatment, septal reduction strategies or surgical myectomy should be considered. Alcohol septal ablation is now the main option for septal reduction therapy [26]. The most common non-fatal complication is atrioventricular block, seen in about 7–20% of patients [27]. The procedure, however, can only be done when the septal anatomy is suitable.

**Fig. 3.**
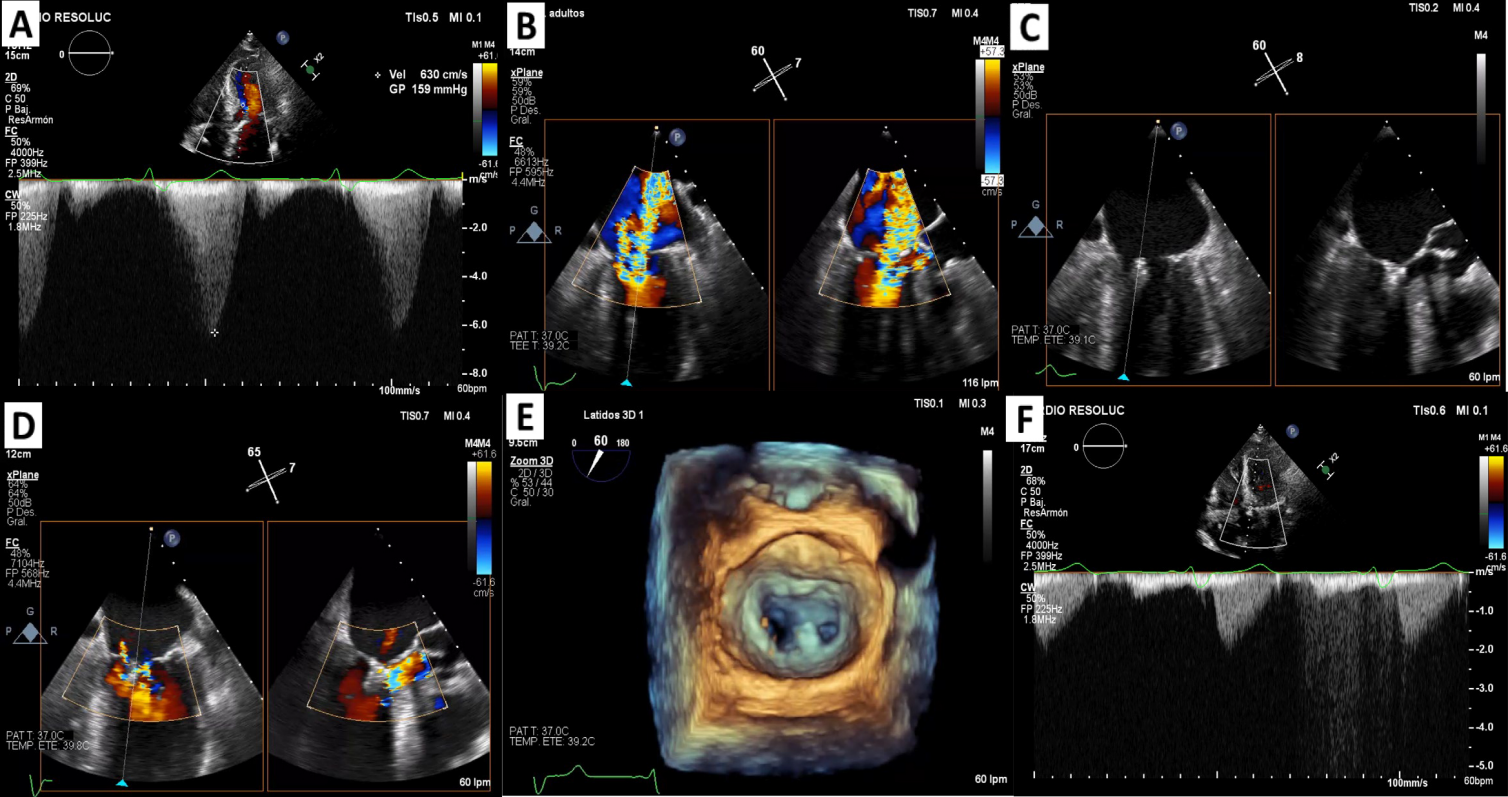
**M-TEER in systolic anterior motion-related mitral regurgitation secondary to HOCM**. (A) Continuous-wave Doppler: LVOT gradient at a rest of 159 mmHg. (B) 2D TEE using orthogonal views: severe mitral regurgitation. (C) Device implantation (MitraClip System) in A3-P3 position. (D) Mild mitral regurgitation post M-TEER. (E) Zoom-3D: final result. (F) Continuous-wave Doppler without LVOT gradient. HOCM, hypertrophic obstructive cardiomyopathy; LVOT, left ventricular outflow tract; TEE, transesophageal echocardiogram; M-TEER, mitral transcatheter edge-to-edge repair. Panels A and F are adapted from our previous work [31] and combined with new data (Panels B–E) generated in this review.

In patients who are not candidates for septal reduction therapies, M-TEER has been explored as a potential alternative to reduce SAM-related LVOT obstruction and MR. Evidence supporting this approach is extremely limited. Sorajja *et al*. [28] conducted a study to evaluate the efficacy of percutaneous mitral valve plication as a therapeutic option for patients with symptomatic HOCM who were not candidates for septal ablation or myectomy. Their report consisted of a small case series including six elderly patients (mean age 83 years, predominantly female), each treated with a single MitraClip device implanted at the A2–P2 scallops. The procedure was technically successful in five patients; the sixth developed cardiac tamponade during the intervention. Significant hemodynamic improvements were observed, including a reduction in LVOT gradient (from 91 ± 44 mmHg to 12 ± 6 mmHg, *p* = 0.007), a decrease in left atrial pressure (from 29 ± 11 mmHg to 20 ± 8 mmHg, *p* = 0.06), a reduction in MR severity (from grade 3.0 to 0.8, *p* = 0.0002), and an increase in cardiac output in four patients (from 3.0 ± 0.6 L/min to 4.3 ± 1.2 L/min, *p* = 0.03). At 1.5 years of follow-up, all patients showed improvement to NYHA functional class ≤II. While early results of percutaneous mitral valve repair were encouraging in this limited population, robust evidence supporting M-TEER in patients with HOCM is still lacking. All available data originate from isolated case reports and very small case series [29, 30], so additional large-scale studies are needed to validate these preliminary results. Therefore, while M-TEER therapy may offer symptomatic improvement in highly selected, elderly, or anatomically unsuitable patients who cannot undergo septal reduction therapy, it should be viewed as an exploratory, off-label strategy rather than a validated alternative to guideline-supported therapies. Fig. 3 (Ref. [31]) shows a patient treated with M-TEER for SAM secondary to HCM.

## 5. Mitral Transcatheter Edge to Edge Repair in Advanced Heart Failure

Advanced HF is associated with a poor prognosis, with annual mortality approaching 20% in de novo HF and a substantial proportion of patients progressing to stage D disease [32]. Secondary MR is common in this population, exacerbating symptoms and outcomes.

Several registries have examined outcomes of M-TEER in patients with advanced HF. The German TRAMI registry included 777 patients and reported on 256 with severely reduced LVEF (<30%) and elevated NT-proBNP. In this subgroup, in-hospital mortality was relatively low (3.1%), one-year mortality reached 24.2%, and nearly 70% of patients improved by at least one NYHA class [33]. Similarly, the EXPAND study, the largest real-world evaluation of the MitraClip NTR/XTR, enrolled over 1000 patients worldwide, including 118 with NYHA IV symptoms [34]. Procedural success was high (92.4%), with sustained MR reduction at 30 days (90.7%) and 12 months (92.9%). Even though mortality and rehospitalizations were more frequent in the NYHA IV group (29.2% vs. 17.7%), functional class and quality of life improved, with more than 70% of patients transitioning to NYHA I/II at one year.

Data from the Italian GIOTTO registry further highlights the prognostic implications of HF severity. Among 984 patients with SMR treated with M-TEER, those fulfilling criteria for advanced HF (NYHA III–IV, LVEF ≤30%, and recent HF hospitalization) had significantly higher two-year mortality (47% vs. 29%). Importantly, achieving an optimal procedural result (MR ≤1+) was associated with a survival benefit in both advanced and non-advanced HF groups, underscoring the critical importance of procedural success [35]. Conversely, hospitalizations for HF remained more frequent in advanced HF despite intervention, illustrating the intrinsic disease progression.

MitraBridge provided complementary evidence in the transplant population. In this multicenter registry, 119 patients with advanced HF and significant MR underwent MitraClip as bridging therapy [36]. Procedural success was 87.5%, 30-day survival was 100%, and one-year survival was 64%. Notably, nearly one-quarter of patients no longer met criteria for listing due to clinical improvement. These findings suggest that M-TEER may provide meaningful stabilization in select patients, delaying or even obviating the need for advanced therapies. However, small retrospective studies evaluating M-TEER prior to left ventricular assist device (LVAD) implantation, including series by Dogan *et al*. [37] and Kreusser *et al*. [38], suggest that although MR reduction is generally achieved, pre-LVAD TEER does not appear to improve survival or halt heart failure progression. These findings highlight the limited impact of M-TEER on long-term outcomes in this population and underscore the complexity of therapy sequencing in patients with advanced HF.

**Fig. 4.**
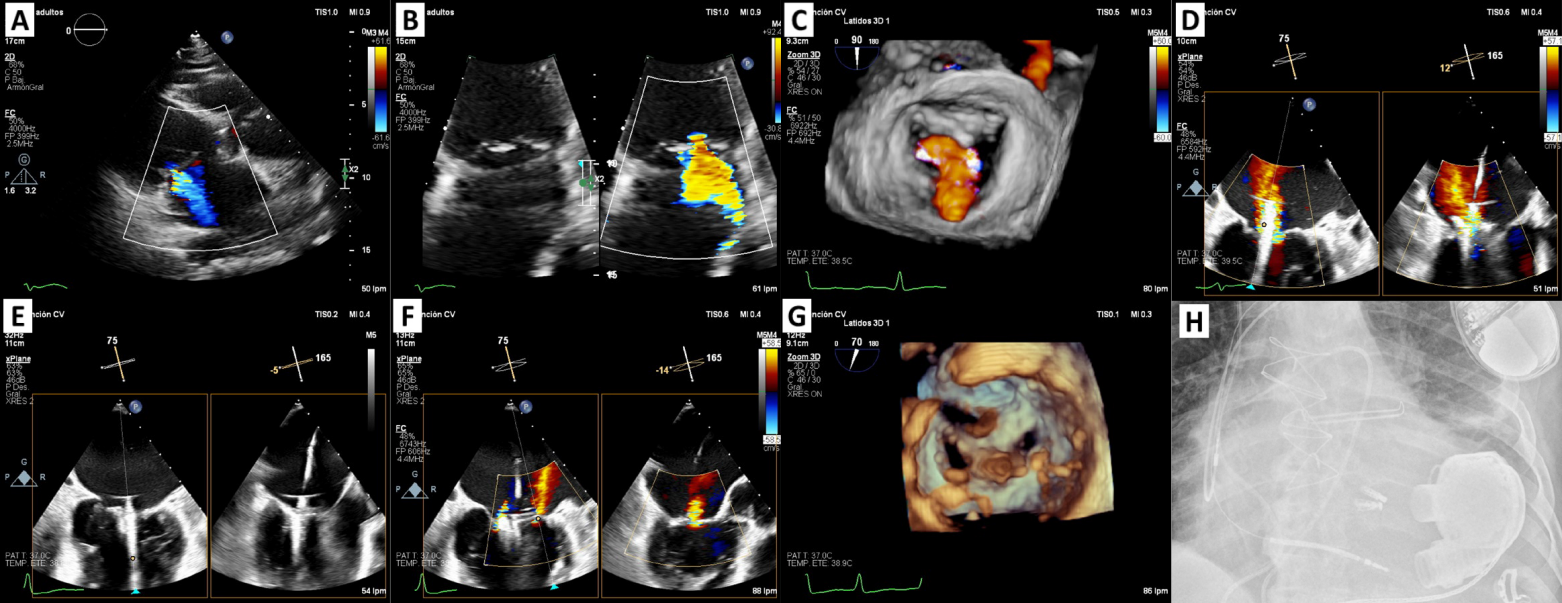
**M-TEER for secondary mitral regurgitation as a bridge to left ventricular assist device in a patient with stage D heart failure**. (A,B) TTE + TEE: secondary severe mitral regurgitation. (C) Zoom-3D with colour: confirms the diagnosis. (D) Device implantation (DragonFly™ System): the first device was implanted in centro-medial position with moderate residual lateral jet. (E) Second device was implanted in a central-lateral position, in close relation to the first one. (F) Mild mitral regurgitation post procedure. (G) Zoom-3D: final result. (H) Fluoroscopy image showed ICD, two DragonFly™ devices and pump housing HM3. M-TEER, mitral transcatheter edge-to-edge repair; TTE, transthoracic echocardiogram; TEE, transesophageal echocardiogram; LVAD, left ventricular assist device; ICD, implantable cardioverter-defibrillator; HM3, HeartMate 3.

Overall, while patients with advanced HF undergoing M-TEER have worse outcomes compared with less advanced cohorts, available evidence demonstrates that the procedure is safe and can provide symptomatic and hemodynamic benefit, particularly when an optimal reduction in MR is achieved. In carefully selected patients, especially those unsuitable for transplant or LVAD, or those requiring stabilization as a bridge to candidacy, M-TEER represents a reasonable therapeutic consideration. Its role should be determined by a multidisciplinary heart team, weighing baseline and anatomical characteristics that predict procedural success, with the understanding that durable advanced therapies continue to offer superior long-term survival (Fig. 4).

## 6. Concomitant Procedures 

Advances in percutaneous therapies have supported the extension of M-TEER toward combined procedures, including concomitant mitral–tricuspid repair, M-TEER performed after or alongside transcatheter aortic valve implantation (TAVI), and integrated strategies with left atrial appendage closure (LAAC). While these approaches appear technically feasible, it is important to acknowledge that the available evidence remains extremely limited and is derived predominantly from observational studies. Available data derive almost entirely from observational series, with inherent risks of selection bias, center effect, and unmeasured confounding. To date, no randomized controlled trials have compared concomitant, staged, or isolated strategies, and therefore no conclusions can be drawn regarding long-term outcomes, patient selection, or comparative benefit. As such, enthusiasm for these techniques should be balanced by the recognition that their role remains exploratory and confined to carefully selected high-risk patients in experienced centers.

**Table:** 

What we know:
- Combined procedures (M-TEER + T-TEER, M-TEER + TAVI, M-TEER + LAAC) are technically feasible and yield high procedural success in published series.
- Early outcomes suggest acceptable safety in carefully selected high-risk patients.
What we don’t know:
- No randomized trials exist comparing combined vs. staged or isolated strategies.
- Long-term durability, impact on survival, and optimal patient selection remain undefined.
- Observational data are subject to selection bias, center-effect, and unmeasured confounding.

### 6.1 Mitral and Tricuspid Valve Repair

In recent years, transcatheter edge-to-edge repair of the tricuspid valve has become the most widely adopted percutaneous treatment for severe tricuspid regurgitation (TR), with procedural success rates consistently >85% and a favorable safety profile [39, 40, 41]. TR is associated with left-sided heart disease in 50% of cases, raising the question of whether combined or staged mitral and tricuspid repair (TMTVR) may improve outcomes compared with isolated intervention [42].

In the comparative analysis of the TRAMI (M-TEER) and TriValve (tricuspid therapies) registries, Mehr *et al*. [43] included 228 patients with severe MR and TR: 106 underwent isolated M-TEER, while 122 received combined mitral + tricuspid repair. At one-year, all-cause mortality was 34% in the mitral-only group versus 16% in the combined group (*p* = 0.035), with multivariable analysis confirming TMTVR therapy as an independent predictor of survival. However, these findings derive from observational registries, and substantial residual confounding cannot be excluded. Causal inference cannot be established.

Further confirmation comes from smaller recent series. Papadopoulos *et al*. [44] reported data in a cohort of 42 patients (median age 77 years, 64% female) undergoing combined M-TEER and T-TEER. Implantation success was 100%, with mean device and procedure times of 39 and 71 minutes, respectively. No major adverse events occurred in-hospital or at 30 days, aside from two cases of tricuspid single leaflet device attachment (4.8%) and one atrial septal defect closure (2.4%). During a median follow-up of 11 months, three patients (7.1%) required hospitalization for HF, but no deaths were reported. At one year, all patients were in NYHA class II or better, MR was ≤2+ in every case, and 81% had only trivial or mild TR.

**Fig. 5.**
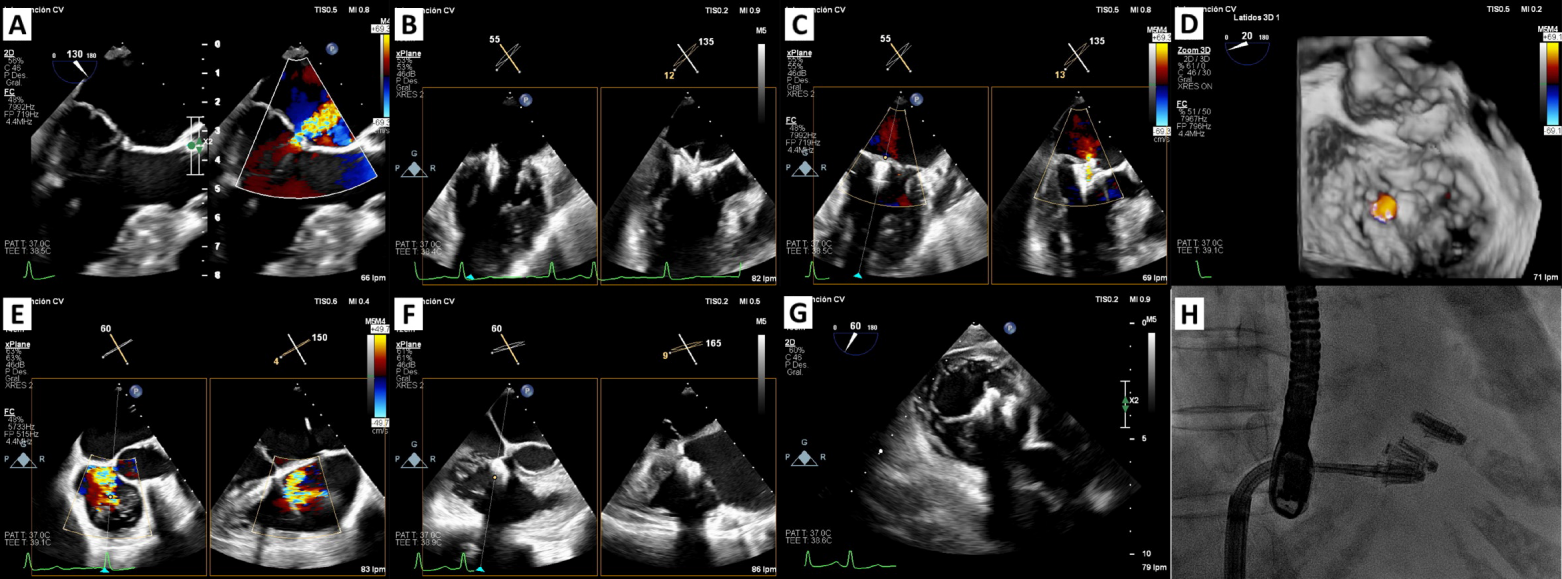
**Concomitant M-TEER and T-TEER**. (A) TEE: central severe mitral regurgitation. (B) Implantation device: Single device (PASCAL P10) in A2-P2 position. (C,D) 2D TEE using orthogonal views (intercommissural and left ventricular outflow tract) and zoom-3D view showing mild mitral regurgitation post procedure. (E) TEE: severe tricuspid regurgitation. (F,G) Implantation devices (PASCAL Ace): the first device was implanted in anteroseptal position and the second device in central anteroseptal position in close relation to the first one. (H) Fluoroscopy image showing all the devices. M-TEER, mitral transcatheter edge-to-edge repair; T-TEER, tricuspid transcatheter edge-to-edge repair; TEE, transesophageal echocardiogram.

Future prospective studies are needed to define the optimal timing and patient selection, including survival, rehospitalizations, right ventricular function, and durability of repair. Until then, concomitant or staged M-TEER and T-TEER (Fig. 5) should be considered on an individual basis, particularly in high-risk surgical candidates with favorable anatomy.

### 6.2 M-TEER and TAVI

The prevalence of at least moderate MR in patients with severe aortic stenosis varies across registries. In the study by Gjini *et al*. [45] among a cohort of 2817 patients with AS, 15% had moderate or severe MR. Similarly, in the PARTNER study, approximately 20% of patients with severe AS undergoing transcatheter or surgical valve replacement presented with concomitant moderate or severe MR [46]. In this context, the role of combined or staged M-TEER is being increasingly explored, although the current evidence remains limited to registries, database analyses, and small series.

One of the largest analyses comes from Zahid *et al*. [47], who used the US Nationwide Readmission Database to evaluate 627 patients treated between 2015 and 2019. Among them, 174 underwent concomitant TAVI + M-TEER, while 453 received a staged approach (TEER after TAVI). In-hospital mortality was similar between groups, but concomitant procedures were associated with longer hospital stays (median 15 vs. 4 days) and a higher rate of non-home discharges. Ando *et al*. [48] reported in a systematic review outcomes in 33 patients (10 studies). In most cases (≈88%) the procedures were staged, with TAVR performed first, while only 12% underwent a truly concomitant intervention [48]. Technical success was achieved in all patients, with post-procedural residual MR reduced to ≤mild in the majority; only two patients had persistent moderate MR despite clip implantation and all-cause mortality occurred in 15% of the population. Complications were infrequent but included pericardial tamponade, stroke, permanent pacemaker requirement, acute kidney injury, and minor bleeding. Altogether, these results highlight the technical feasibility and safety of simultaneous dual-valve interventions in carefully selected patients. Nevertheless, current data remain limited to registries and observational cohorts, with no randomized controlled trials directly comparing concomitant versus staged strategies.

### 6.3 M-TEER and Left Atrial Appendage Closure

Atrial fibrillation frequently coexists with severe MR, contributing to symptom burden and the need for long-term anticoagulation [49]. In this setting, combining M-TEER with LAAC in a single intervention has been proposed as a therapeutic strategy, as it addresses both the valvular pathology and stroke prevention simultaneously and may spare many patients from long-term anticoagulant therapy. Both procedures share venous access and transseptal puncture, potentially avoiding repeat anesthesia and additional hospitalizations. The rationale for a combined approach is partly extrapolated from surgical experience, in which LAAC performed at the time of mitral surgery was associated with lower thromboembolic risk, as shown in LAAOS III [50]. However, these data derive from a different clinical and procedural context and cannot be directly translated to transcatheter interventions.

To date, no randomized trials have evaluated the combination of M-TEER and LAAC. Available evidence is limited to small observational studies and registries involving highly selected patients; antithrombotic regimens varied widely across studies, and long-term stroke prevention remains unproven. Small feasibility series by Freixa *et al*. [51] and Kuwata *et al*. [52] reported success rates above 90% with very few complications. Subsequent multicenter experiences reported similar technical success. In D’Amico’s registry of 30 patients, procedural success was 93% [53], while the GRASP subanalysis by Frazzetto *et al*. [54] found that among 41 patients, outcomes at one year were similar to isolated M-TEER but bleeding events were markedly lower (0% vs 19%). The prospective WATCH-TEER registry of 24 patients also showed high procedural success, effective MR reduction, and no major procedural complications, with most patients able to discontinue anticoagulation during follow-up [55]. When pooling the evidence from 11 published series including 139 patients, procedural success averaged 96% with very low rates of serious complications such as device embolization, pericardial effusion, or thromboembolism [56].

Altogether, these results suggest that performing M-TEER and LAAC together is not only feasible but also safe, and may offer particular benefit for elderly, frail patients with severe MR, atrial fibrillation, and contraindications to long-term anticoagulation. Despite these findings, the existing evidence is based on small, nonrandomized cohorts and is prone to selection bias, with limited follow-up and heterogeneous antithrombotic regimens. Larger registries and, ideally, randomized trials will be needed to confirm whether combining M-TEER and LAAC translates into superior long-term outcomes compared with staged or isolated procedures.

## 7. Mitral Annulus Calcification

Mitral annulus calcification (MAC) is a chronic, degenerative process characterized by calcium deposition within the fibrous support ring of the mitral valve. It may range from focal, mild calcification to extensive circumferential involvement extending into the leaflets, subvalvular apparatus, or ventricular myocardium. MAC is frequent among older patients with MR and is associated with increased procedural complexity and higher baseline risk. Contemporary series show that M-TEER is feasible and generally safe in carefully selected MAC anatomies, but outcomes depend heavily on detailed multimodality imaging.

**Favorable anatomy typically includes:**

- calcium confined to the annulus without extension into the coaptation zone;

- predominantly posterior (rather than circumferential) distribution;

- absence of bulky calcification protruding into the LV inflow;

- preserved leaflet mobility without marked thickening or restriction;

- posterior leaflet length ≥7 mm;

- absence of large flail segments (flail gap >10 mm or width >15 mm);

- acceptable predicted transmitral gradients (interpreted in the context of body size, cardiac output, and baseline mitral valve area), and adequate distance between the calcified annulus and the papillary muscles.

**Conversely, “no-go” features for M-TEER include:**

- extensive leaflet calcification limiting grasping;

- significant calcification in the coaptation line;

- circumferential or bulky annular calcification protruding toward the LVOT;

- severe leaflet tethering or markedly reduced mobility;

- Predicted post–M-TEER transmitral gradients are considered prohibitive after individualized assessment of flow conditions, body size, and baseline risk of mitral stenosis.

- concomitant LVOT calcification compromising device trajectory.

In the multicenter study by Fernández-Peregrina *et al*. [57] (61 MAC vs 791 no/mild MAC), procedural success was similar (91.8% vs 95.1%; *p* = 0.268) with low complication rates. At 1 year, MR ≤2+ was achieved in 90.6% (MAC) and 79.5% (no/mild MAC), and ~80% in both groups were NYHA ≤II [57]. There was a trend toward higher all-cause mortality in MAC (19.7% vs 11.3%; *p* = 0.050), while cardiovascular mortality did not differ significantly, underscoring that excess risk in MAC may relate to overall substrate rather than valve failure per se.

Larger multicenter cohorts and meta-analyses align with these findings. Hatab *et al*. [58] reported that M-TEER in significant MAC achieved similar 1-year MR reduction compared with none/mild MAC, but moderate/severe MAC carried higher 1-year mortality and less symptomatic improvement, reinforcing the prognostic weight of the underlying disease. A recent meta-analysis likewise found durable MR reduction and functional gains after M-TEER in MAC, yet higher 1-year mortality persisted, pointing to patient risk rather than procedural futility [59].

Interestingly, Condos *et al*. [60] reported the feasibility of M-TEER in 13 patients with MAC and large mitral annuli as a preparatory step before transcatheter mitral valve replacement. The rationale was to shorten the intercommisural distance using M-TEER. Commissural M-TEER was successful in all patients, with no leaflet detachment, and NTW devices were the most frequently employed. Following TMVR, paravalvular leak was absent or limited to trace in all cases. Overall, M-TEER can be a reasonable option in patients with MAC when the anatomy suggests that a durable repair is achievable—meaning adequate leaflet coaptation and acceptable transmitral gradients. The decision, however, has to be made in the context of the patient’s overall risk profile and expected benefit. Careful imaging, using echocardiography and CT when necessary, together with discussion in a multidisciplinary heart team, is key to weighing the potential gains of the procedure against the challenges and risks imposed by MAC.

## 8. Conclusions 

Mitral transcatheter edge-to-edge repair has evolved into a versatile and increasingly adopted therapy across a broadening spectrum of clinical scenarios beyond current guideline indications. Evidence consistently supports its safety and effectiveness in high-risk patients with acute post-myocardial infarction MR, moderate secondary MR, advanced heart failure, HOCM, mitral annulus calcification, and in carefully selected candidates for combined structural interventions. Across these settings, successful MR reduction is associated with meaningful symptomatic improvement and, in several scenarios, with improved survival compared with conservative therapy. Nevertheless, patient selection remains the cornerstone of optimal outcomes, and the balance between anatomical feasibility, procedural success, and long-term benefit must be individualized within a multidisciplinary heart team. Future progress will rely on randomized trials clarifying the role of M-TEER in high-risk patients, establishing its utility in advanced HF as bridge or destination strategy, and evaluating combined transcatheter approaches and interventions in MAC. Such studies are essential to refine patient selection, determine long-term durability, and delineate the true boundaries of expanding indications for M-TEER.

## Abbreviations

HF, heart failure; HOCM, hypertrophic obstructive cardiomyopathy; LV, left ventricle; LVEF, left ventricular ejection fraction; MR, mitral regurgitation; M-TEER, mitral transcatheter edge-to-edge repair; AMI, acute myocardial infarction; NYHA, New York Heart Association; PMR, papillary muscle rupture; GDMT, guideline-directed medical therapy; LAAC, left atrial appendage closure; TAVI, transcatheter aortic valve implantation; MAC, mitral annulus calcification; LVAD, left ventricular assist device.
